# Investigation of D_1_ Receptor–Agonist Interactions and D_1_/D_2_ Agonist Selectivity Using a Combination of Pharmacophore and Receptor Homology Modeling

**DOI:** 10.1002/cmdc.201100546

**Published:** 2012-02-07

**Authors:** Marcus Malo, Lars Brive, Kristina Luthman, Peder Svensson

**Affiliations:** [a] NeuroSearch Sweden ABArvid Wallgrens Backe 20, 413 46 Göteborg (Sweden); [b] Department of Chemistry, Medicinal Chemistry, University of Gothenburg412 96 Göteborg (Sweden); [c] Department of Biomedicine, University of GothenburgP.O. Box 440, 405 30 Göteborg (Sweden)

**Keywords:** dopamine agonists, GPCRs, pharmacophore modeling, protein structure modeling, selectivity

## Abstract

The aim of this study was to use a combined structure and pharmacophore modeling approach to extract information regarding dopamine D_1_ receptor agonism and D_1_/D_2_ agonist selectivity. A 3D structure model of the D_1_ receptor in its agonist-bound state was constructed with a full D_1_ agonist present in the binding site. Two different binding modes were identified using (+)-doxanthrine or SKF89626 in the modeling procedure. The 3D model was further compared with a selective D_1_ agonist pharmacophore model. The pharmacophore feature arrangement was found to be in good agreement with the binding site composition of the receptor model, but the excluded volumes did not fully reflect the shape of the agonist binding pocket. A new receptor-based pharmacophore model was developed with forbidden volumes centered on atom positions of amino acids in the binding site. The new pharmacophore model showed a similar ability to discriminate as the previous model. A comparison of the 3D structures and pharmacophore models of D_1_ and D_2_ receptors revealed differences in shape and ligand-interacting features that determine selectivity of D_1_ and D_2_ receptor agonists. A hydrogen bond pharmacophoric feature (Ser-TM5) was shown to contribute most to the selectivity. Non-conserved residues in the binding pocket that strongly contribute to D_1_/D_2_ receptor agonist selectivity were also identified; those were Ser/Cys^3.36^, Tyr/Phe^5.38^, Ser/Tyr^5.41^, and Asn/His^6.55^ in the transmembrane (TM) helix region, together with Ser/Ile and Leu/Asn in the second extracellular loop (EC2). This work provides useful information for the design of new selective D_1_ and D_2_ agonists. The combined receptor structure and pharmacophore modeling approach is considered to be general, and could therefore be applied to other ligand–protein interactions for which experimental information is limited.

Supporting information for this article is available on the WWW under http://dx.doi.org/10.1002/cmdc.201100546.

## Introduction

The dopamine receptors belong to the G-protein-coupled receptor (GPCR) superfamily. They are membrane proteins with seven transmembrane helices (TM1–7), and are involved in second-messenger signal-transduction cascades via the guanine binding proteins (G proteins). Based on their structure, pharmacology, and transduction pathways, the dopamine receptors are grouped into two subfamilies: the D_1_-like (D_1_ and D_5_) and the D_2_-like (D_2_, D_3_, and D_4_) receptors. D_1_-like receptors couple to the G protein G_s_ and stimulate adenylate cyclase, which catalyzes the conversion of ATP to cyclic AMP, whereas the D_2_-like receptors inhibit this enzyme via the G proteins G_i_ and G_o_.^1^

Dopamine receptor agonists have seen extensive clinical use in the treatment of Parkinson's disease (PD). For example, dopamine itself, administered as its biosynthetic precursor l-DOPA, has been in use for more than four decades. The mixed D_1_/D_2_ receptor agonist apomorphine,^2^, ^3^ as well as the orally bioavailable D_2_ agonists bromocriptine, pergolide, pramipexole, and ropinirole, have all been shown to be useful in the treatment of PD.^4^ The D_2_ receptor agonists in clinical use have far lower efficacy than l-DOPA, and may also cause side effects such as nausea and psychotic symptoms that are associated with D_2_ receptor activation. The critical role of D_1_ receptors in the treatment of PD was discovered in the 1980s, with the selective agonists SKF38393^5^ and CY-208-243.^6^ These drugs have only modest therapeutic effect, but because both are partial agonists, they led to the hypothesis that the beneficial antiparkinsonian activity depends on efficacy at D_1_. Interestingly, the full and highly selective D_1_ receptor agonist ABT-431 (prodrug of A86929) has been shown to have the same antiparkinsonian efficacy as l-DOPA, with decreased neurological side effects.^7^ In addition, Blanchet et al.^8^ reported that the well-known full D_1_ agonist dihydrexidine (DHX) also shows a definite motor improvement in patients with PD, but with a narrow therapeutic window.


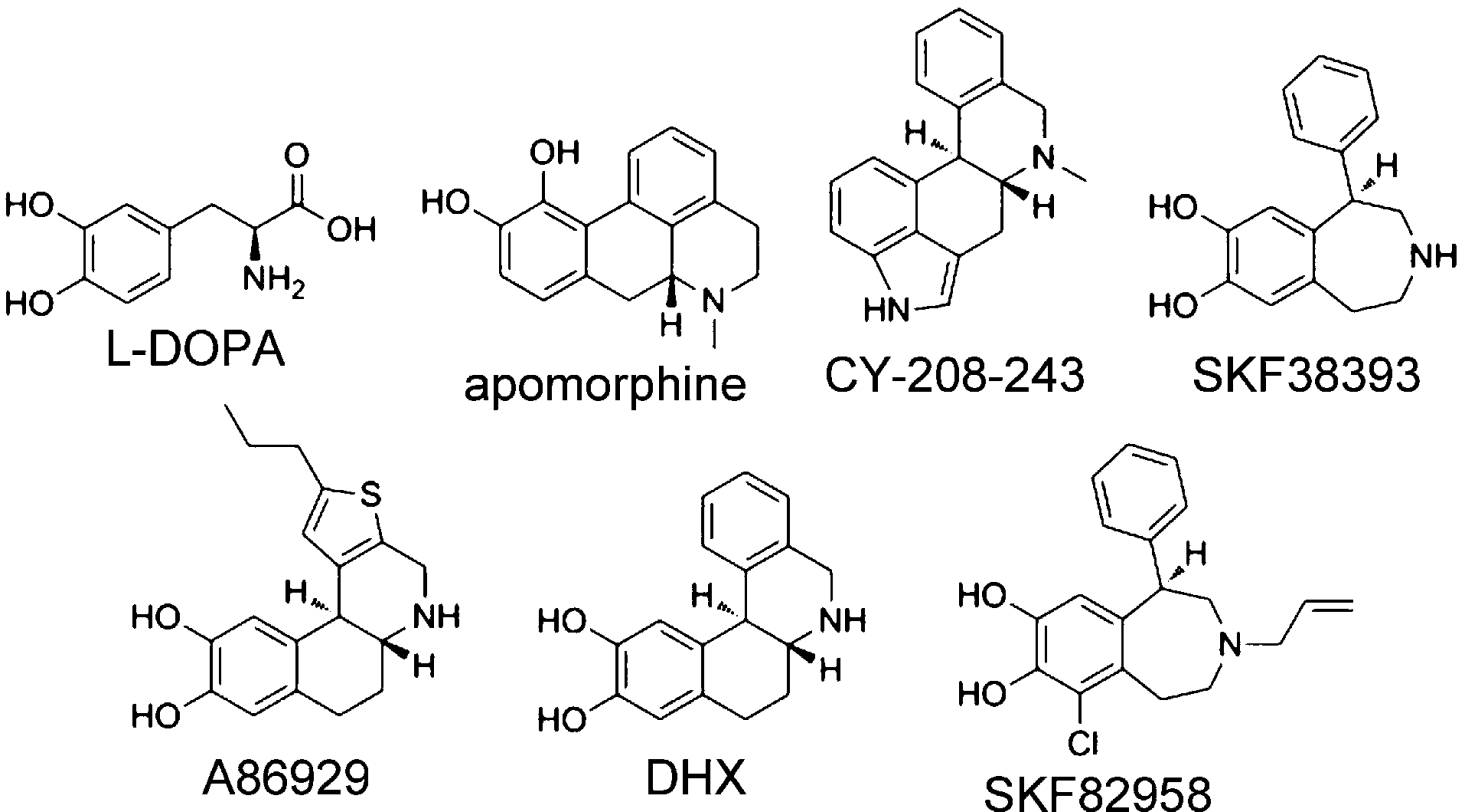


The detailed mechanism underlying D_1_ receptor activation is yet unknown, but extensive site-directed mutagenesis^9^–^11^ and fluorescence^10^, ^12^ studies have indicated the amino acid residues that are involved in the activation of GPCRs. The well-conserved D(E)R^3.50^Y^1^ motif at the intracellular side of transmembrane helix 3 (TM3) is highly involved in receptor activation. In site-directed mutagenesis studies on the α_1b_^9^ and β_2_^10^, ^11^ adrenergic receptors, for example, it has been shown that the interaction of TM3 with an acidic residue in TM6 stabilizes the inactive receptor state. This “ionic lock” restrains the location of TM6, as also shown for bovine^14^ and squid^15^ rhodopsin. In addition, mutation to the uncharged glutamine in D(E)RY in rhodopsin results in a constitutively active opsin receptor.^16^

Agonists that bind GPCRs have been suggested to be associated with the transition of helix conformations from the inactive to active state.^17^–^19^ D_1_ and D_2_ receptor agonists share the dopamine three-point pharmacophore: a hydrogen bond accepting/donating feature, an aromatic ring, and an amino function. Generally, the selective D_1_ agonists are larger than the D_2_ agonists and contain either primary or secondary amino functions. In contrast, propyl substituents on the amine are favored in several D_2_ agonists, as the D_2_ receptor has a propyl binding pocket.^20^ To the best of our knowledge, there is no full D_1_ agonist that lacks the catechol motif, whereas there are several D_2_ agonists known to have only a single hydroxy group or other hydrogen bond accepting/donating functions. In addition, Payne et al.^21^ have shown that the non-hydroxylated dipropylaminotetralin analogue (*S*)-DPAT is a full D_2_ agonist, but with lower affinity than the hydroxylated agonist analogues.

It has been shown that D_1_ and D_2_ receptors are co-localized as hetero-oligomers, both in the striatum and in the cortex.^22^ The oligomeric receptors have a synergistic effect, and selective activation of either one of the receptors results in co-internalization of the hetero-oligomeric complex. It has also been shown that D_1_ internalization efficacy is independent of both the structural class and the affinity of the agonists.^23^, ^24^ However, although there is functional selectivity between the agonist activation of adenylate cyclase and internalization, it seems that only agonists with high efficacy can mediate internalization.^24^

In the present study we developed dopamine D_1_ receptor models to better understand the molecular basis for selectivity between full agonists and structurally similar inactive compounds. We focused on characterizing the binding site for agonists using available published binding selectivity data^25^–^28^ and mutation data.^29^, ^30^ Dopamine D_1_ receptor structure models with all loops except the third intracellular loop (IC3) were built by using the structure of the human β_2_ adrenergic receptor (adrb2; PDB code: 2RH1) as template. The protein structure models were compared and combined with the selective dopamine D_1_ pharmacophore model published recently by our group.^31^ The pharmacophore model was refined further based on the binding pocket of the dopamine D_1_ receptor (drd1) structure model. The drd1 model was compared with the previously published drd2 structure model^31^ to study the features that determine the selectivity between D_1_ and D_2_ receptor agonists. With this combined pharmacophore and receptor modeling approach, we can make optimal use of all available structure–activity relationship (SAR), mutational, and structural information to gain a more detailed understanding of D_1_ agonism and agonist selectivity between D_1_ and D_2_ receptors.

## Important amino acids for agonist binding to the D_1_ receptor

The D_1_ dopamine receptor is not as well characterized as the D_2_ or the β_2_ receptors, but like all catecholamine receptors it contains an aspartic acid residue in TM3 (Asp103^3.32^), which forms a salt bridge with the basic amino group of the ligands.^32^ According to Pollock et al., the D_1_ receptor also includes a cluster of conserved serine residues (Ser198^5.42^, 199^5.43^, and 202^5.46^) in TM5, of which Ser198^5.42^ contributes mainly to the binding of dopamine and the partial agonist SKF38393.^29^ It was shown that the binding of dopamine and SKF38393 to a Ser198^5.42^→Ala mutated receptor decreased more than 50- and 14-fold, respectively. The study also included data on an additional benzazepine derivative, the full agonist SKF82958 (fenoldopam), which together with SKF38393 is negatively affected by a Ser199^5.43^→Ala mutation (5- and 13-fold, respectively). Dopamine is also affected considerably by this mutation (10-fold), but on the other hand, it is even more sensitive to a Ser202^5.46^→Ala mutation. In a functional assay, SKF38393 showed greater potency toward the Ser202^5.46^→Ala mutant than toward wild-type, while the maximum intrinsic activity was decreased,^29^ indicating that Ser202^5.46^ is highly important for efficacy. The study also included two structurally related monohydroxylated benzazepines that act as antagonists; these compounds are completely insensitive to the Ser202^5.46^→Ala mutation.^13^ These functional results indicate that the catechol motif is essential for agonism and crucial for full D_1_ agonism, and that an interaction between catechol and Ser202^5.46^ is required for receptor activation. The D_1_ receptor has one additional serine residue (Ser197^5.41^) in the same region, but it is most likely oriented toward the membrane and thereby not accessible for direct ligand binding.

Tomic et al.^30^ investigated dopamine binding to drd1 with two double mutations in the orthosteric binding site (Ser199^5.43^→Val/Ser202^5.46^→Ala and Cys106^3.35^→Ala/Ser107^3.36^→Gly). As expected, the serine double mutation results in a drastic loss in binding affinity. However, dopamine binding affinity was also decreased (sixfold) with the other double mutation. The Ser107^3.36^ residue is located one turn down in the membrane relative to Asp103^3.32^. The corresponding serine mutation (Ser^3.36^→Ala) in the 5-HT_2B_ receptor resulted in a 30-fold decrease in binding affinity for 5-HT. However, the potent 5-HT receptor partial agonist LSD is unaffected by the mutation.^33^, ^34^ Almaula et al.^33^ suggested that the amino function of 5-HT interacts with both Ser^3.36^ and Asp^3.32^, whereas LSD is sterically hindered to form these simultaneous interactions. In addition, an alternative mutation at this position (Ser^3.36^→Cys) decreased the binding of 5-HT, but not as much as did the Ser^3.36^→Ala mutation. Ser^3.36^ is conserved between 5-HT_2A/2B_ and the D_1_ receptor, whereas the D_2_ receptor has a cysteine residue at this position. In drd1 there is an additional amino acid (Thr108^3.37^) in TM3 that is accessible for ligand binding. According to site-directed mutagenesis studies on Thr^3.37^ in the adrenergic α_1B_ receptor (ada1b), this residue, together with Ser^3.36^, is important for receptor activation.^35^

No mutation studies involving the hydrophobic amino acids in TM6 of drd1 are available. However, because this motif is well conserved among the GPCRs it can be expected that the D_1_ agonists make similar interactions with these amino acids as do agonists of other monoaminergic receptors. Based on mutational studies on drd2^36^ and SAR data, in combination with modeling of the α_2A_ adrenergic receptor,^37^ we expect that Phe289^6.52^ in drd1 interacts via a face-to-edge π–π interaction with the aromatic catechol function of the agonist.^36^, ^37^ In addition, the hydrophobic residues Trp285^6.48^ and Phe288^6.51^ are expected to be accessible for ligand interaction, in analogy with the D_2_ receptor.^38^ Asn^6.55^ is conserved between drd1 and the template structure (adrb2) and has been shown to be important for agonist binding in adrenergic β_2_ receptors.^39^ This asparagine residue is believed to bind to the β-OH group of adrb2 agonists.^39^ Although D_1_ agonists do not contain this function, Asn292^6.55^ may still be important for agonist recognition, either directly or indirectly. Manivet and co-workers^34^ demonstrated that Asn^6.55^ in the 5-HT_2B_ receptor is involved in direct or indirect 5-HT binding, while Ser^5.43^ (Ser199^5.43^ in D_1_) is not. This is in agreement with mutational studies on the D_1_ receptor, where the endogenous ligand is less affected by this specific mutation than by other mutations in the serine cluster.^29^

**Figure 1:**
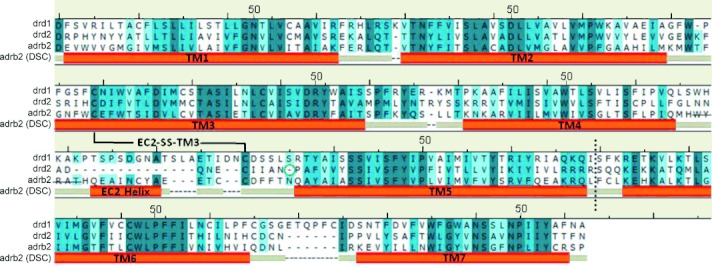
The refined multiple sequence alignment of the human adrenergic β_2_ receptor (adrb2, 2RH1) and the dopamine D_1_ (drd1) and D_2_ (drd2) receptors. The adrb2 (DSC) bars indicate the transmembrane (TM) helix regions and the second extracellular loop helix (EC2 Helix) in the adrb2 structure. The lysozyme in adrb2 and the third intracellular loop (IC3) in drd1 and drd2 between TM5 and TM6 were excised; this is indicated with a dashed line. The strikethrough amino acid stretch WYRAT was cut out in the template structure. The green ring at the N terminus of TM5 in the drd2 sequence indicates the gap caused by the smaller number of amino acids between the cysteine bridge (EC2-SS-TM3) and TM5. Amino acids marked in dark blue indicate fully conserved positions, medium blue residues have highly similar physicochemical character, and light blue residues have less similar physicochemical character. The conserved cysteine bridge between TM3 and EC2 (EC2-SS-TM3) is indicated. The most conserved residue in each helix is marked with the index 50.

As in all monoaminergic receptors, drd1 has a disulfide bridge that connects Cys186 in the extracellular loop 2 (EC2) with Cys96^3.25^ in TM3 (EC2-SS-TM3); this constrains the loop on top of the binding site crevice. The β_2_ adrenergic receptor (adrb2) and the dopamine D_1_ receptor (drd1) have an equal number of amino acids in the stretch between EC2-SS-TM3 and TM5, which is one more than the number of residues present in the corresponding stretch in drd2. This makes the portion of EC2 closest to the ligand binding site less constrained than in drd2, which may, therefore, allow larger substituents to point toward the extracellular side of the receptor. In addition, the amino acid sequences differ significantly in the stretch (CDSSLS, drd1; CIIAN-, drd2; Figure 1) between the disulfide bridge and TM5, which may be crucial contributors to the D_2_/D_1_ receptor agonist selectivity.

## Results and Discussion

### Multiple sequence alignment and manual adjustments

A multiple sequence alignment of the human adrenergic β_2_ (adrb2, PDB code: 2RH1), drd1, and drd2 was performed by using Clustal W (version 2.0.11).^40^ The program predicted the alignment of the first five helices correctly, but not TM6 and TM7, as the third cytoplasmic loop in drd2 is considerably longer than in adrb2. The removal of the third intracellular loop (IC3) in drd2 allowed a satisfactory alignment of the remaining conserved helical regions (TM6–7; see alignment in Supporting Information figure 1).

The binding pocket is defined by amino acids within 3.5 Å of the D_1_ receptor agonist doxanthrine, which was used as environment^2^ during the homology modeling procedure. The obtained Clustal W alignment was carefully checked in the non-conserved positions close to the binding site and in loop regions. Manual adjustments in some parts of the sequence alignments were made with the purpose of improving the final homology model (Figure 1). The following adjustments were made:

TM3: The sequence PFG in drd1 in EC1 is moved toward the N terminus of TM3 to fill a gap in that region.
TM4 and EC2: The amino acid stretch WYRAT (strikethrough in Figure 1) between TM4 and the EC2 helix in adrb2 is cut out in the template to allow the longer loop in the drd1 model to find a more reasonable conformation. In addition, the C terminus of TM4 in adrb2 contains weak helix-forming amino acids (MH), whereas the corresponding amino acids in D_1_ (LS) are stronger helical formers. The removal of the WYRAT stretch in the template thus allowed the program to freely predict the secondary structure at the extracellular end of TM4.

The sequence similarity between adrb2 and drd1 in the manually adjusted alignment was 36 % in total, 43 % in the TM helix region, and 67 % in the binding pocket (the corresponding sequence similarity between drd1 and drd2 is 34, 43, and 55 %, respectively).

### D_1_ receptor homology modeling

The D_1_ homology model was built with the high-resolution β_2_ receptor structure (2RH1) as template. Modeling was performed with the MOE software package^41^ (version 2009.10) using the Amber99^42^ force field with an R-field solvation model. See reference 31 for methodology details.

### A proposed D_1_ receptor–agonist model and comparison with the published selective D_1_ agonist pharmacophore model

The D_1_ homology model was developed in a similar manner as described in the preceding paper for the construction of the D_2_ model.^31^ During generation of the homology model, the potent and full D_1_ agonist (+)-doxanthrine^26^ (DOX) was present in the binding site. DOX is a chromane-based analogue of dihydrexidine (DHX), but unlike DHX, it is selective for D_1_ receptors.^26^ Twenty structure models were generated, and for each model the side chain conformations were sampled three times to give a total of 60 models. The backbone structure of the generated drd1 models differed considerably in the C-terminal part of TM4, in the EC2 close to the disulfide bridge, and around the IC1 loop, but particularly in EC3. The amino acid side chain conformations also differed in these regions. Such differences were also observed in helical regions where more than one optimal packing solution is possible.

The structure quality of the receptor models was evaluated with the MOE evaluation features.^41^ For example: 1) bond lengths, bond angles, and dihedral angles of the protein backbone; 2) Ramachandran plots of ϕ–ψ dihedrals (General, Glycine, Proline and pre-Proline [for explanations see plot for the final model in figure 2 of the Supporting Information]); and 3) side chain rotamer quality. The focus was directed toward the binding site region and the important agonist key interacting amino acids. One dopamine D_1_ receptor homology model with desirable geometry was selected for further preparation. Hydrogen atoms were added to the ligand, and the ionization and tautomeric states of the ligand–receptor complex were determined. The complex was refined further by energy minimization with DOX present in the binding site with motion restrictions on all heavy atoms. This step was followed by an unconstrained energy minimization. The final model was subjected to detailed analysis and comparison with the recently published D_1_ agonist pharmacophore model generated in our research group.^43^

**Figure 2:**
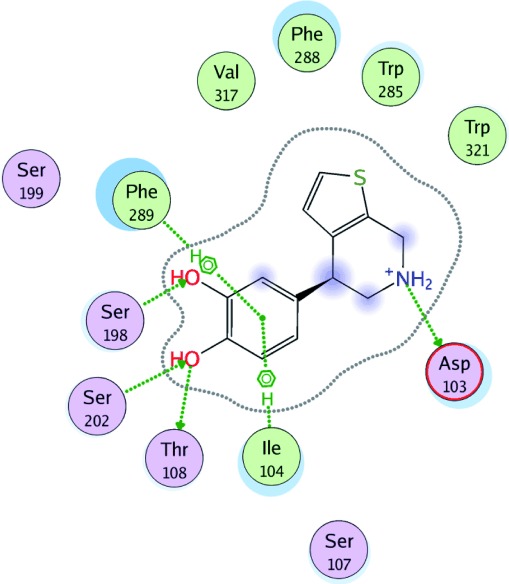
Schematic view of the interactions between the full agonist SKF89626 and the dopamine D_1_ receptor homology model. The typical catecholamine agonist–receptor key interactions with Asp103^3.32^, Ser198^5.42^, and Ser202^5.46^ are shown. The *meta*-hydroxy group of SKF89626 interacts via hydrogen bonding with Ser198^5.42^, and the *para*-hydroxy group interacts with Ser202^5.46^. In addition, the *para*-hydroxy also forms a hydrogen bond to Thr108^3.37^. Phe289^6.52^ forms a face-to-edge π–π interaction with the agonist, and a methyl–π interaction with Ile104^3.33^ is formed as well. Polar residues are shown in purple, whereas hydrophobic residues are in green. Blue shades indicate ligand–receptor solvent accessibility.

**Figure 3:**
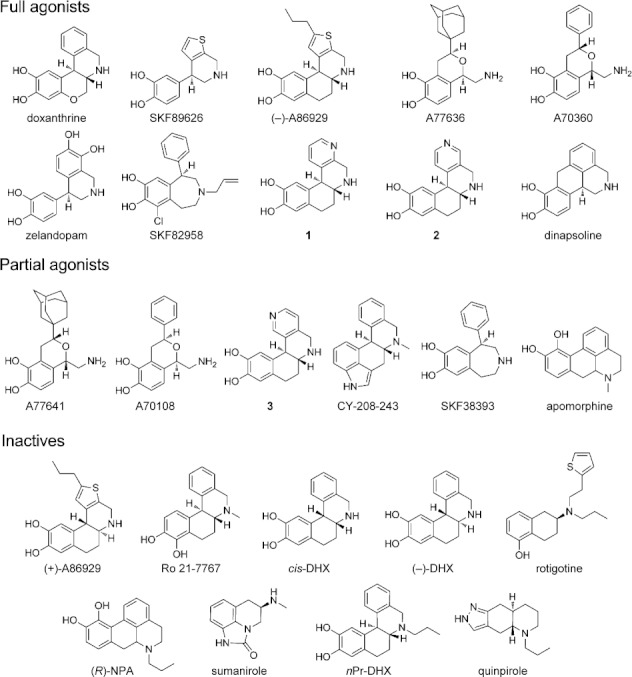
Selected full and partial D_1_ receptor agonists and structurally similar inactives screened against the new protein structure based pharmacophore model. For a more detailed account of the set, see reference 43.

The geometry of the key interacting pharmacophore features was in good agreement with the positions of the corresponding interacting amino acids in the homology model. In contrast to the D_2_ case, good alignment of the pharmacophore feature (Ser-TM5) with the serine cluster of the receptor was obtained.^31^ The excluded volumes, however, did not fully reflect the shape of the binding site. A new version of the pharmacophore with protein structure-based positioning of the excluded volumes was constructed. When introducing the excluded volumes of the new D_1_ pharmacophore model we discovered that the D_1_ agonists may bind in two distinct binding modes involving two different receptor binding site conformations. The ligands SKF89626, a super-agonist with an intrinsic activity of 120 %^44^ (Figure 2) and zelandopam (Figure 3) have similar scaffolds, in which the catechol ring is linked via a single bond to a bicyclic motif. All full D_1_ agonists that we found in the literature contain a catechol function, and when these aromatic rings are aligned, the binding mode of SKF89626^45^ and zelandopam^46^ differ from the remaining set of agonists. The low-energy conformations of these agonists have the bicyclic motif oriented perpendicular to the catechol. In evaluating the rotational energy of the dihedral angle between the ring systems in SKF89626, we found that the energy barrier to planarity was ∼8 kcal mol^−1^ (Supporting Information figure 3). In an initial pharmacophore screen of the set of ligands, SKF89626 and zelandopam clashed into excluded volumes corresponding to amino acids in TM6. Dinapsoline (Figure 6 below) also had steric clashes in this region. Therefore, different side chain rotamers of Phe288^6.51^ were evaluated to create space for dinapsoline, SKF89626, and zelandopam. An energy minimization of the DOX-generated D_1_ homology model with SKF89626 present in the binding site resulted in the movement of Val317^7.39^ toward Phe288^6.51^ and Trp285^6.48^.

A subsequent unconstrained energy minimization retained this conformation. This modification of the model does not affect the binding mode of DOX. The exact positions of features, relative to the new set of structure-based excluded volumes, were optimized based on pharmacophore hit rate of actives and inactives.

The typical monoaminergic key interactions, that is, the ionic interaction to Asp103^3.32^, the hydrogen bonds to the serine residues (Ser198^5.42^ and Ser202^5.46^) and the face-to-edge π–π interaction with Phe289^6.52^, are present in the D_1_ receptor–agonist complexes (Figure 2). The distance from the oxygen atom in the *para*-hydroxy group of SKF89626 to the oxygen in the hydroxy group of Ser202^5.46^ is 2.7 Å, and the O-H-O(Ser202^5.46^) angle is 178°. In addition, the *para*-hydroxy group forms a hydrogen bond with Thr108^3.37^ (*d* = 2.7 Å, ∢=176°). The oxygen atom in the corresponding *meta* position interacts with Ser198^5.42^ with a distance of 2.8 Å between the heavy atoms and an O-H-O(Ser198^5.42^) angle of 166°.

Ser199^5.43^ is 4.6 Å away from the oxygen in the *meta*-hydroxy group of SKF89626 and therefore does not interact directly with the ligand, but instead forms a hydrogen bond with Asn292^6.55^. This is in agreement with the findings by Pollock and co-workers^29^ that agonist binding is least sensitive for mutations of Ser199^5.43^ in the serine cluster. The basic amino group of the ligand interacts almost symmetrically with both oxygen atoms in Asp103^3.32^ and forms a salt bridge with N—O distances of 2.7 and 2.9 Å, and N-H-O(Asp103^3.32^) angles of 158° and 130°. Ser107^3.36^, which was shown to be important for agonist binding in both the drd1^30^ and 5-HT_2A_ receptors,^33^ is directed toward the binding crevice and interacts with the backbone carbonyl of Asp103^3.32^. One of the hydrogen atoms in the amino function of SKF89626 is just outside the defined distance to form a hydrogen bond with Ser107^3.36^ (*d* = 4.6 Å, ∢=175°). Phe289^6.52^, which has proven to be important for agonist binding and activation of GPCRs,^36^, ^37^ forms a face-to-edge π–π interaction with the catechol motif of the ligand (Figure 2). In addition, the position of the thiophene moiety of SKF89626 is stabilized by the aromatic/hydrophobic cluster in TM6 and TM7, which includes residues Phe288^6.51^, Phe289^6.52^, Trp285^6.48^, Val317^7.39^, and Trp321^7.43^ (Figure 2). Two of the amino acids in EC2 (Ser188 and Leu190), which were found to be important for agonist binding and activation of GPCRs,^47^, ^48^ are directed downward into the binding site crevice and can thus make additional interactions with the ligand.

### Evaluation of the selected D_1_ agonist-induced receptor model

The final SKF89626-minimized D_1_ receptor model showed good structural quality (Ramachandran plots are shown in figure 2 of the Supporting Information) and had an RMSD in relation to the template structure of 2.78 Å for Cα and 1.94 Å for Cα of the TM region. The volume of the binding pocket is 495 Å^3^. The model was investigated and evaluated further using the Procheck program.^49^ With the exclusion of glycines and prolines, 86 % of the residues belonged to the most favored region of the Ramachandran map, 16 % in the allowed, and 1 % in the generously allowed region according to Procheck. No residues belonged to disallowed regions. All main chain and side chain geometries were designated to the “better” class. Eight close contacts were identified, all between the receptor and the ligand, of which five included hydrogen atoms involved in hydrogen bonds and one from the face-to-edge π–π interaction between Phe289^6.52^ and the ligand. Close contacts are defined as pairs of non-bonded atoms within a distance of 2.6 Å from one another.

### Refinement of the D_1_ pharmacophore model

**Figure 4:**
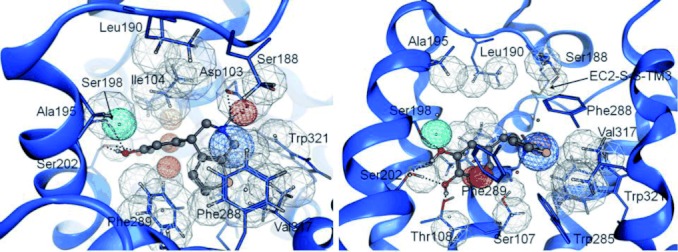
Top (left) and side view (right) of the new receptor-based pharmacophore model superimposed onto the D_1_ structure model. The transmembrane helix 6 (TM6) and the hydrogen atoms of the interacting amino acids, together with the corresponding excluded volumes, are not shown. The conformation of SKF89626 is taken from the ligand–receptor model complex, while the relative positions of the pharmacophore features are tuned to generate the best hit rate.

The excluded volumes of the D_1_ pharmacophore model were rearranged in the same way as for the D_2_ pharmacophore model,^31^ and were based on the shape of the agonist binding pocket. Thus, excluded volumes were introduced over the hydrogen atoms in amino acids that are located within 3 Å of the ligand, including those involved in intermolecular hydrogen bonding. Due to the discovery of the two different binding modes, two ligand–receptor complexes, SKF89626 and DOX, were used to identify the excluded volume positions. The initial radii of the excluded volumes were selected from the van der Waals radii (vdWr) proposed by Bondi,^50^ (i.e. 1.2 Å for aliphatic and 1.0 Å for aromatic, hydroxy, and amine [polar] hydrogen atoms). The sizes of the excluded volumes were tuned manually until the pharmacophore model was sufficiently discriminating. The final radii were 1.5 Å for aliphatic and 1.3 Å for aromatic and polar hydrogen atoms. Excluded volumes covering the aromatic rings in aromatic amino acid residues were introduced to account for face-to-edge clashes between aromatic ring systems (Figure 4). The center of the volume is located at the center of mass of the ring (*r* = 2.5 Å). The alignment of the feature part of the pharmacophore model in relation to the set of new excluded volumes derived from the receptor model was tuned manually and evaluated by the hit rate of the ligand training set. The model providing the best discriminating ability between actives and inactives was selected. The pharmacophoric features do not superimpose perfectly with the positioning of SKF89626, but the orientation of the features is still in good agreement with the key interacting amino acids (i.e., AspTM3, SerTM5 and Aro superimposed with Asp103^3.32^, Ser198^5.42^, and Phe289^6.52^, respectively, in the receptor model; Figure 4). The new refined pharmacophore model was screened against two conformational ensembles of D_1_ ligands that were generated with both MMFF(S)^51^ (MOE)^41^ and OPLS^52^ (MacroModel)^53^ force fields, using Born solvation (water). The initial screen of the new pharmacophore model based on the drd1 receptor model showed similar results as those obtained with the previously published pharmacophore model^31^ (Table 1, structures shown either in Figure 3 or in reference 31).

**Table 1:** Pharmacophore model search results from the new and old D_1_ agonist pharmacophore models using two different conformational ensembles of the set of active and inactive ligands.

		New pharmacophore model						Old pharmacophore model					
Ligand		MOE stochastic search Born solvation MMFF94(S)[a]			MacroModel serial torsion search GB/SA solvation OPLS2005[b]			MOE stochastic search Born solvation MMFF94(S)[a]			MacroModel serial torsion search GB/SA solvation OPLS2005[b]		
		Δ*E*[c]	RMSD[d]	#c/#h[e]	Δ*E*[c]	RMSD[d]	#c/#h[e]	Δ*E*[c]	RMSD[d]	#c/#h[e]	Δ*E*[c]	RMSD[d]	#c/#h[e]
Doxanthrine	Full	0.5	0.60	2/1	0.0	0.11	14/14	0.0	0.12	2/2	0.0	0.11	14/6
SKF89626	Full	0.1	0.61	4/2	0.0	0.63	20/6	0.1	0.61	4/2	0.0	0.63	20/6
(−)-A86929	Full	0.0	0.54	10/6	0.0	0.52	73/58	0.0	0.54	10/10	0.5	0.12	73/36
A77636	Full	1.4	0.30	3/1	0.0	0.26	11/7	0.0	0.28	3/2	0.0	0.26	11/7
A70360	Full	1.5	0.65	7/1			13/0	0.0	0.67	7/5	2.1	0.67	13/5
Zelandopam	Full			4/0	0.0	0.63	40/13			4/0	2.9	0.63	40/2
SKF82958[f]	Full	3.8	0.47	22/1	2.2	0.40	50/6	1.8	0.41	22/1	0.0	0.43	50/8
**1**	Full	1.6	0.59	2/1	1.6	0.61	6/2	0.0	0.22	2/2	0.0	0.13	6/4
**2**	Full	0.0	0.23	2/2	0.2	0.62	12/6	0.0	0.23	2/2	0.0	0.12	12/6
Dinapsoline	Full	0.0	0.39	1/1	0.0	0.45	4/4	0.0	0.39	1/1	0.0	0.45	6/4
Dopamine	Full	0.0	0.66	6/1	0.0	0.64	20/8	0.0	0.66	6/1	0.0	0.64	20/8
DHX	Full	0.8	0.63	2/1	0.0	0.13	14/14	0.0	0.23	2/1	0.0	0.13	14/8
**4**[g]	Full	0.0	0.55	2/2	0.0	0.56	16/8						
4-OH-Dinapsoline[g]	Full	0.0	0.36	1/1	0.0	0.46	8/5						
6-Et-Dinapsoline[g]	Full	0.0	0.37	6/3	0.0	0.46	16/16						
A77641	Partial			3/0			11/0			3/0	2.8	0.67	11/2
A70108	Partial			6/0	0.0	0.56	11/7	0.0	0.71	6/3	0.0	0.72	11/7
**3**	Partial	0.0	0.62	2/2	0.9	0.61	8/4			2/0			8/0
CY-208-243	Partial			3/0			1/0			3/0			1/0
SKF38393	Partial	0.4	0.68	5/2	0.0	0.47	20/16	0.0	0.43	5/3	0.0	0.62	20/10
Apomorphine	Partial	0.0	0.61	2/1	0.0	0.62	4/2	0.0	0.61	2/1	0.0	0.62	4/2
(+)-A86929	inactive			10/0			41/0			10/0			49/0
Ro 21-7767[f]	Inactive			4/0			8/0			4/0			8/0
*cis*-DHX	Inactive			5/0			12/0	2.7	0.62	5/1	3.4	0.62	12/4
(−)-DHX	inactive	0.8	0.74	2/1			8/0	0.8	0.74	2/1			8/0
Rotigotine[f]	inactive	1.3	0.73	186/5	1.9	0.72	486/13	3.6	0.69	186/2			486/0
(*R*)-NPA[f]	Inactive	2.5	0.60	6/1			27/0			6/0			27/0
Sumanirole	Inactive			5/0			4/0			5/0			4/0
*n*Pr-DHX[f]	inactive	1.3	0.42	17/2	3.4	0.43	56/2			17/0			56/0
Quinpirole	Inactive			4/0			9/0			4/0			9/0
**5**	inactive			2/0			8/0						

[a] The energy cutoff for conformations generated in MOE is 4 kcal mol^−1^.

[b] The energy cutoff for conformations generated in MacroModel is 16.7 kJ mol^−1^ (∼4 kcal mol^−1^).

[c] The lowest relative energy [kcal mol^−1^], with respect to the most stable conformer in the ensemble, for the conformers that fit the pharmacophore model.

[d] Root of the mean square distance between the center of the pharmacophore features and their matching ligand annotation points.

[e] #c: number of conformations generated for the respective method; #h: number of conformations that hit the pharmacophore model.

[f] The amine is tertiary and therefore considered chiral, and two different configurations have been used in the modeling.

[g] The compounds are new in this study and therefore were not screened against the previously published pharmacophore model.

### Evaluation of the D_1_ agonist pharmacophore model

The set of D_1_ ligands used in the previously published pharmacophore modeling study,^43^ supplemented with some novel compounds described in detail below (15 full and 6 partial agonists, and 10 structurally similar inactives; Figure 3) were screened against the new protein structure based pharmacophore model. Of the OPLS-generated set of ligand conformations, all but A70360 of the full agonists (14/15), and all but two (4/6) of the partial agonists (A77641 and CY-208-243) fit into the model. In addition, the model excluded all except two (2/10) of the inactives (rotigotine and *n*Pr-DHX; Table 1). The D_2_-selective agonist *n*Pr-DHX, which is defined as inactive in this study, shows low D_1_ receptor affinity (IC_50_=651 nm, EC_50_>10^4^ nm) and moderate efficacy (36 %).^27^ The best hit of *n*Pr-DHX adopts a conformation with a relative energy of 3.4 kcal mol^−1^. All other ligands that hit the pharmacophore have a best-hitting conformation with lower relative energy than *n*Pr-DHX. The hit conformation of the D_1_ inactive ligand rotigotine had a relative energy of 1.9 kcal mol^−1^ and an RMSD of 0.72, which is the highest value of all pharmacophore hits. In addition, the number of hits relative to the number of conformations was low: 2/56 for *n*Pr-DHX and 13/486 for rotigotine.

**Figure 5:**
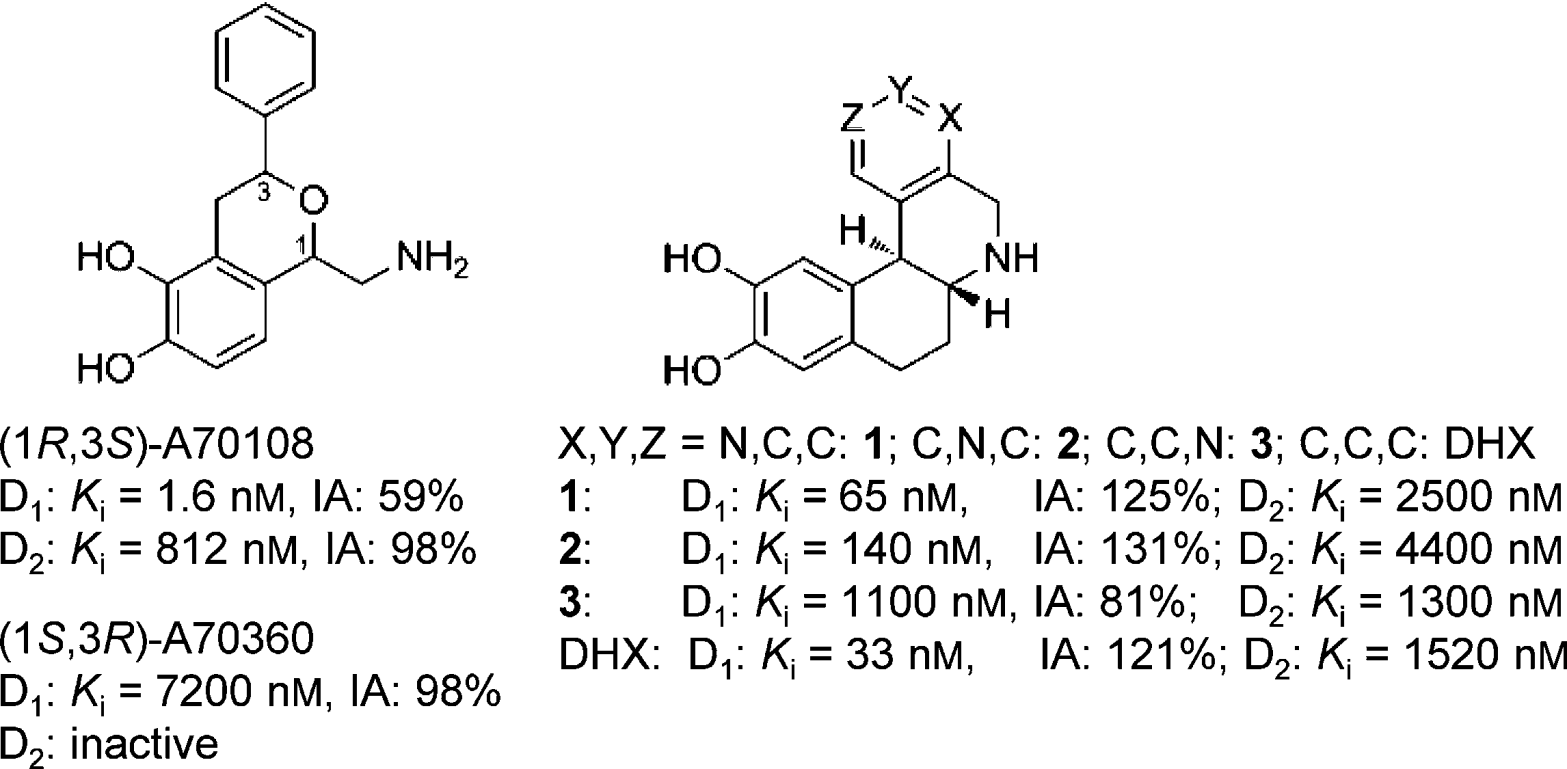
The potent partial D_1_ agonist A70108 (left) together with its enantiomer, the full but less potent agonist A70360. DHX and its three aza analogues **1**–**3** are shown at right.

To further evaluate the model, we screened the pharmacophore model against the same ligand set, but the conformations were generated with MMFF(S), which resulted in two more pharmacophore hits of the inactives ((*R*)-NPA and *cis*-DHX), but with unfavorable energy, hit rate, and RMSD (Table 1). In addition, one more active ligand was excluded: zelandopam. Interestingly, one conformation of the active ligand A70360 fit the pharmacophore with a high relative energy, whereas none of the conformations of its enantiomer A70108 did (Figure 5 A). This is in contrast to the OPLS-generated conformers, where A70108 fit, but A70360 was excluded. The reason why they are on the border of matching the pharmacophore model may be reflected by the fact that A70360 is a full agonist, but with only low affinity, whereas its enantiomer A70108 is a potent partial agonist (IA=60 %).^25^

Other notable results are the differences in receptor interactions made by the DHX aza analogues **1**–**3** (Figure 5) in the D_1_ receptor model,^28^ for which the nitrogen atom in the most potent and full agonist **1** may form a hydrogen bond with Ser188 in EC2 (Supporting Information figure 4). The distance between the heavy atoms in the hydrogen bond is 4.3 Å and the N-H-O(Ser188) angle is 139°, which is not considered to be an optimal hydrogen bond. However, because Ser188 is located in the loop it is more flexible, and therefore able to change its conformation; otherwise a water molecule may mediate the interaction. Analogue **2** has slightly less affinity than **1**, but shows full efficacy for the D_1_ receptor. Finally, analogue **3** has even lower affinity and decreased efficacy as well. In the pharmacophore hits of **3**, the nitrogen atom points toward the hydrophobic Leu190 residue located in EC2. Interestingly, **3** is the most potent analogue at the D_2_ receptor with a similar affinity as DHX.^28^ The corresponding residue to Leu190 in drd2 is an asparagine (Asn186), whereas the Ser188 in drd1 corresponds to Ile184, which may reflect the D_1_/D_2_ binding selectivity of the aza analogues.

**Figure 6:**
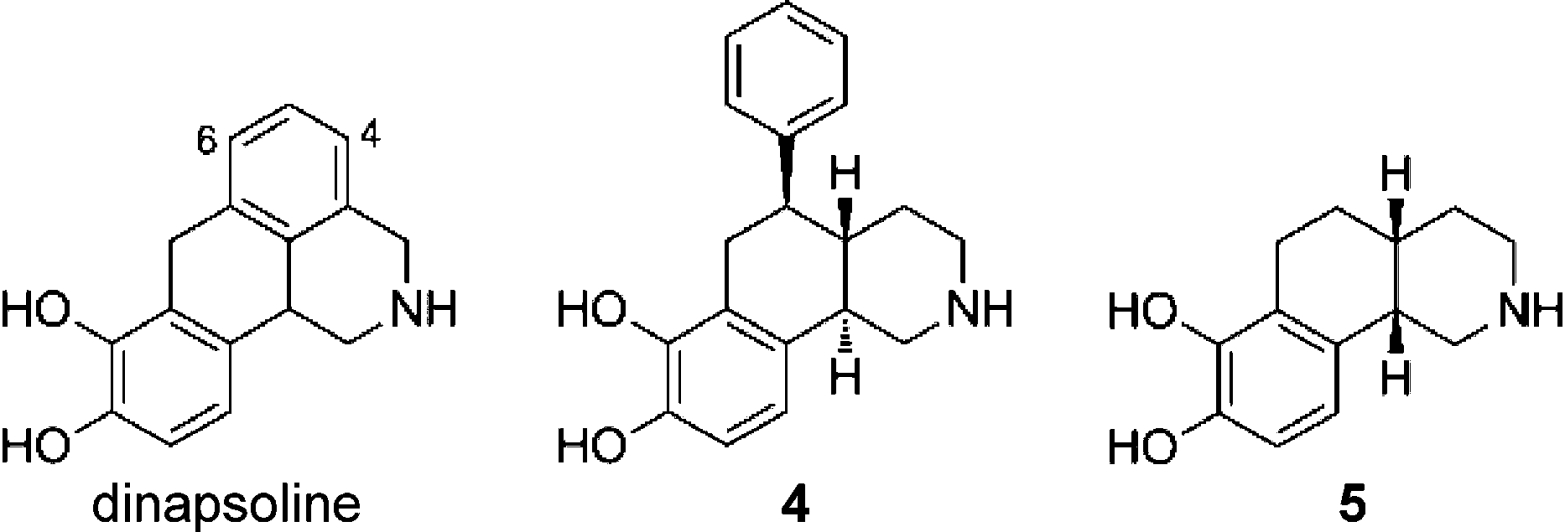
The agonist dinapsoline together with the octahydrobenzo[*h*]isoquinoline analogues **4** and **5**; compound **4** is a potent full D_1_ agonist, and **5** is inactive.

Sit et al.^54^ studied dinapsoline analogues and discovered that the 4-OH- and 6-Et-dinapsoline analogues (Figure 6) showed a similar affinity as dinapsoline, but the 6-Et analogue had enhanced intrinsic activity. Bonner et al.^55^ recently published a novel series of octahydrobenzo[*h*]isoquinolines, of which the most potent compound (**4**, Figure 6) shows higher affinity and similar efficacy for the D_1_ receptor than do DHX and doxantrine. The *cis* analogue that lacks the phenyl group, compound **5**, is considered inactive and is discriminated by the pharmacophore model, whereas compound **4** matches nicely (Table 1).

### Comparison of the dopamine D_1_ and D_2_ receptor structure models and corresponding agonist pharmacophore models

**Figure 7:**
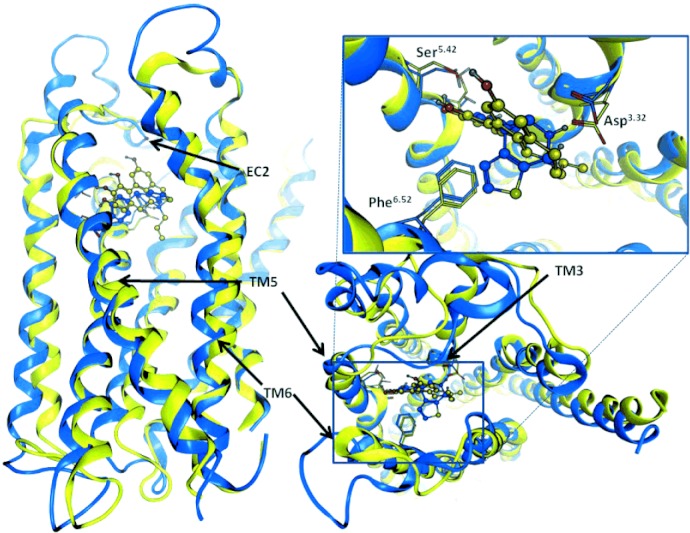
Two orthogonal views of the dopamine D_1_ (blue) and D_2_ (yellow) receptor models together with the corresponding full agonists (*R*)-2-OH-NPA (blue) and SKF89626 (yellow) present in their binding sites. The typical monoaminergic key interacting amino acid residues are shown explicitly. The structures differ particularly in the second and the third extracellular loops (EC2 and EC3), but also in the transmembrane (TM) region, where important interacting amino acids are positioned.

The D_1_ homology model has an RMSD in relation to the corresponding D_2_ model of 2.2 Å for Cα and 1.4 Å for Cα in the transmembrane region. Thus, the dopamine receptor models are more similar to each other in the TM region than they are to the template structure (drd1–adrb2: 2.8 Å for all Cα and 1.9 Å for Cα in the TM region; drd2–adrb2: 2.1 Å for all Cα and 1.5 Å for Cα in the TM region). An overlay of the two models is shown in Figure 7. The orthosteric binding pocket, defined as amino acids within 3.5 Å of the corresponding agonists, is located between TM2, 3, 5, 6, and 7, whereas EC2 lines the top of the binding crevice. The volume of the binding pocket of drd1 is 495 Å^3^, whereas it is considerably smaller (371 Å^3^) for drd2. The radii of the optimized excluded volumes of the pharmacophore models are 1.5 Å for aliphatic and 1.3 Å for aromatic and polar hydrogen atoms in drd1, and respectively 2.1 and 1.9 Å in drd2. This indicates that the models probably underestimate the actual size difference between the receptor binding sites.

The optimized positions of features, relative to the new set of structure-based excluded volumes in the D_1_ pharmacophore model, are shifted downward, corresponding to a deeper binding mode in the receptor compared with the corresponding D_2_ model. This is in agreement with the involvement of Ser^3.36^ in D_1_ agonist binding, as discussed above. There are 22 amino acids that constitute the binding site, 10 of which are not conserved between the receptors and thus could be expected to contribute to agonist selectivity.

**Figure 8:**
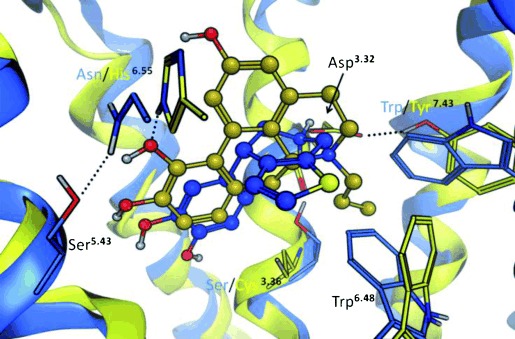
Side view of the superposed dopamine D_1_ (blue) and D_2_ (yellow) receptor models. Transmembrane helix 6 (TM6) is cut out. The conserved tryptophan residue in TM6 that differs in conformation, together with the non-conserved amino acids in TM3, TM6, and TM7, are included and colored by corresponding receptor. Together with the amino acids in TM2, TM3, and TM7, Trp^6.48^ forms the D_2_-characteristic propyl pocket, which is a major contributor to D_1_/D_2_ selectivity. Tyr^7.43^ in drd2 interacts with Asp^3.32^, whereas the corresponding residue (Trp^7.43^) is unable to make that bond and is instead rotated toward TM6 and Trp^6.48^. His^6.55^ interacts with the *meta*-hydroxy group of the D_2_ agonist (*R*)-2-OH-NPA (yellow), while the corresponding Asn^6.55^ forms a hydrogen bond with Ser^5.43^. The D_1_ agonist SKF89626 (blue) binds deeper in the binding crevice and makes interactions with both Ser^3.36^ and Asp^3.32^.

Next to the conserved Asp^3.32^, drd2 has a valine residue, whereas drd1 has a slightly bulkier isoleucine. One turn down relative to Asp^3.32^, a serine residue (Ser107^3.36^) is positioned in drd1 and a cysteine (Cys118^3.36^) in drd2. Cysteine has an inferior hydrogen bonding capacity to that of serine, and hydrophilic elements are therefore less favorable in D_2_ ligands (such as the DHX analogue DOX). DOX is a selective potent D_1_ agonist, and it contains an ether function that points toward Ser/Cys^3.36^. In our drd1 receptor model there is a hydrogen bond between Ser107^3.36^ and the backbone carbonyl in Asp103^3.32^, whereas Cys118^3.36^ is rotated into the drd2 binding pocket and prevents the deeper binding mode (Figure 8). The D_2_ agonist pharmacophore model has a forbidden volume (ExclO) in this region, which excludes oxygen atoms and thereby prevents DOX from matching the model. In addition, the majority of the D_1_ agonists contain a primary or secondary amine which can more easily form hydrogen bonds with Ser107^3.36^ without steric clashes. Almaula et al.^33^ suggested that the amino function of 5-HT interacts with both Ser^3.36^ and Asp^3.32^ in the 5-HT_2A_ receptor, whereas the partial agonist LSD has a tertiary amino function and is thereby sterically hindered from forming these simultaneous interactions. This may be the reason for the absence of full D_1_ agonists with tertiary amino groups. There is one exception—SKF82958—which has an allyl substituent on the amine. Interestingly, the *N*-methyl-substituted analogue is a partial agonist, whereas the secondary amine analogue is a full agonist. SKF82958 has a chlorine atom in the *meta* position of the aromatic ring which has been shown to enhance both affinity and efficacy.^23^

In TM5 the serine residues that are part of the binding site are conserved between drd1 and drd2, but one turn toward the extracellular side of the receptor, drd1 has a tyrosine and drd2 a phenylalanine residue. TM5 in the drd2 model is rotated slightly inward toward the ligand binding site relative to the drd1 model, which makes the serine residues in drd2 less optimally positioned for catechol interaction. This is also reflected in the D_2_ agonist pharmacophore model, in which the hit rate was retained when the Ser-TM5 feature was redefined from essential to optional. In the D_1_ pharmacophore model the Ser-TM5 feature must be essential for retaining the hit rate.

Both receptors contain a highly conserved hydrophobic face in TM6, but they differ in one important position, where drd2 has a histidine and drd1 an asparagine residue, the latter being conserved with the template structure. In mutagenesis studies His393^6.55^ has been shown to be highly involved in agonist binding in drd2, but the corresponding Asn292^6.55^ in drd1 has not been studied. As described above, Asn293^6.55^ in adrb2 has been shown to be involved in agonist binding, and in the drd2 model His393^6.55^ is accessible for ligand interaction, whereas Asn292^6.55^ in drd1 is rotated toward TM5 and interacts with Ser199^5.43^ (Figure 8). The additional polar interaction with His393^6.55^ in the D_2_ receptor may be one reason for D_2_ agonism with aromatic substitutions other than the catechol function. D_1_ agonists can only make polar interactions in this region with the TM5 serines, and for that a catechol function seems to be optimal. The phenylalanine residue (Phe^6.52^), which forms a face-to-edge π–π interaction with the agonists, is positioned almost identically in the two models. The rotamers of the tryptophan residue (Trp^6.48^) one turn down from Phe^6.51^ relative to the membrane differ to some extent between the receptors. Trp386^6.48^ in drd2 is rotated in the direction toward TM7. Trp^6.48^ in drd1 cannot adopt the same conformation as in drd2 due to steric hindrance of the non-conserved Trp321^7.43^ residue in TM7, which is rotated out toward the ligand (Figure 8). The corresponding Tyr416^7.43^ in the D_2_ receptor interacts with Asp114^3.32^ and is rotated toward TM2.

**Figure 9:**
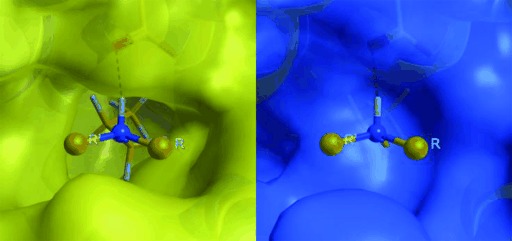
Representation of the solvent-accessible surface of the D_2_ (left) and D_1_ (right) receptors, as viewed from the binding pocket in the direction of the D_2_-characteristic propyl pocket region. The *N*-propyl functional group of (*R*)-2-OH-NPA is included to illustrate the shape difference between the receptors.

The rotation gives rise to a cavity referred to as the propyl pocket, which is localized between the residues Val83^2.53^, Cys118^3.36^, Trp386^6.48^, Thr412^7.39^, and Tyr416^7.43^. This cavity is not present in the drd1 model (Figure 8 and 9).^31^ The geometrical differences in TM6 and TM7 between D_1_ and D_2_ may be the reason why we see two different binding modes for D_1_ agonists and only one for D_2_ agonists (Figure 8).

The second extracellular loop (EC2) differs considerably between the two dopamine receptor models and is also most likely a major contributor to D_1_/D_2_ agonist selectivity. The EC2 in drd1 is more flexible than in drd2 due to the longer amino acid chain between the cysteine bridge (EC2-SS-TM3) and TM5. This could explain the larger substituents in the D_1_ agonists in the direction toward the extracellular side. Another reason for selectivity could be the switch in polarity of the two amino acids in EC2 pointing downward into the binding crevice (Leu190 in drd1 corresponds to Asn186 in drd2, and Ser188 in drd1 corresponds to Ile184 in drd2; Figure 8 and Supporting Information figure 4). We suggest that the extended EC2 stretch in combination with a deeper binding mode in drd1 is the reason for the larger average size of D_1_ agonists relative to D_2_ agonists.

## Conclusions

A 3D structure model of the D_1_ receptor was developed and compared with our previously published selective D_1_ agonist pharmacophore model.^31^ The pharmacophore model was aligned and compared with the receptor model. The positions of the pharmacophore features were in agreement with the D_1_ agonist key interactions identified in the receptor model and were supported by mutation data. Furthermore, a new refined pharmacophore model guided by the shape of the binding site in the receptor model was developed. The pharmacophore and the protein structure models were constructed based on structural information together with binding and mutation data. The combined modeling approach helps to identify strengths and weaknesses in both models. The D_1_-selective and potent agonist doxanthrine was positioned in the binding site during construction. The 3D structure model was modified slightly to allow an alternate agonist binding mode. The 3D structure model of the receptor showed good geometric quality, and the typical dopamine receptor agonist key interactions were present.

The D_1_ agonist pharmacophore and the receptor models were compared with the corresponding D_2_ agonist models to identify differences and thereby pinpoint reasons behind D_1_/D_2_ receptor agonist selectivity. We suggest that the D_1_ agonists bind deeper in the binding site, which may be a consequence of interactions with Ser107^3.36^. The serine residue is positioned one turn down from Asp^3.32^, which forms a salt bridge with the amino function in the agonist. Interactions between Ser107^3.36^ and tertiary amino functions in D_1_ receptor agonists might be difficult, owing to steric hindrance. The selective drd2 agonists often have a propyl-substituted tertiary amino function, with the propyl group fitting well into a hydrophobic region present in the D_2_ receptor binding pocket (Figure 9).

The combined pharmacophore and receptor modeling approach enabled optimal use of all relevant data on receptor subtype selectivity, such as SAR, mutational, protein structure, and sequence data for each receptor subtype. This approach has provided a strong basis for the interpretation of the requirements for dopamine D_1_/D_2_ selectivity based on what is known in the field to date.

## Footnotes

0 To facilitate a comparison between different GPCRs, we use the indexing method introduced by Ballesteros and Weinstein,^13^ where the most conserved residue in every transmembrane (TM) helix is given the index number 50. For example, the Arg residue in the highly conserved DRY motif at the cytoplasmic end of TM3 is denoted Arg^3.50^; the other residues in TM3 are then indexed relative to this position, with the previous residue as Asp^3.49^, and the subsequent as Tyr^3.51^. In addition, the absolute number of each residue in the amino acid sequence is included. The index number 50 for each helix in the D_1_ receptor is highlighted in Figure 1.

0 The environment atoms (for example ligand structures and/or structural water molecules) are specified by the user and are included in the homology modeling procedure.

## Supporting information

Detailed facts of importance to specialist readers are published as ”Supporting Information”. Such documents are peer-reviewed, but not copy-edited or typeset. They are made available as submitted by the authors.
