# Supplementary material for: Investigation of D1 Receptor–Agonist Interactions and D1/D2 Agonist Selectivity Using a Combination of Pharmacophore and Receptor Homology Modeling
Source: ChemMedChem. 2012 Feb 7;7(3):483–94. doi: 10.1002/cmdc.201100546 (PMC3382191; doi:10.1002/cmdc.201100546)
Supplement: Supplementary file 1 [file cmdc0007-0483-SD1.pdf]

CHEMMEDCHEM
Chemistry & Drug Discovery

Supporting Information

© Copyright Wiley-VCH Verlag GmbH & Co. KGaA, 69451 Weinheim, 2012

Investigation of D_1 Receptor–Agonist Interactions and D_1/D_2 Agonist Selectivity Using a Combination of Pharmacophore and Receptor Homology Modeling

Marcus Malo,[b] Lars Brive,[c] Kristina Luthman,[b] and Peder Svensson*[a]

cmdc_201100546_sm_miscellaneous_information.pdf

Supporting Information

```
CLUSTAL 2.0.11 multiple sequence alignment


drd1_human       -MRTLNTSAMDG-----------TGLVVERDFSVRILTACFLSLLILSTLLGNTLVCAAV 48
drd2_human       -MDPLNLSWYDDDLERQNWSRPFNGSDGKADRPHYNYYATLLTLLIAVIVFGNVLVCMAV 59
adrb2_human      MGQPGNGSAFLLAPNRS--HAPDHDVTQQRDEVWVVGMGIVMSLIVLAIVFGNVLVITAI 58
                    . * *                .   : *       . .::*::   ::**.**  *:

drd1_human       IRFRHLRSKVTNFFVISLAVSDLLVAVLVMPWKAVAEIAGFWPFG-SFCNIWVAFDIMCS 107
drd2_human       SREKALQT-TTNYLIVSLAVADLLVATLVMPWVVYLEVVGEWKFSRIHCDIFVTLDVMMC 118
adrb2_human      AKFERLQT-VTNYFITSLACADLVMGLAVVPFGAAHILMKMWTFGNFWCEFWTSIDVLCV 117
                  : . *:: .**::: *** :**::.  *:*: .   :   * *.   *:::.::*::

drd1_human       TASILNLCVISVDRYWAISSPFRYERKMTPK-AAFILISVAWTLSVLISFIPVQLSWHKA 166
drd2_human       TASILNLCAISIDRYTAVAMPMLYNTRYSSKRRVTVMISIVWVLSFTIS-CPLLFGLNNA 177
adrb2_human      TASIETLCVIAVDRYFAITSPFKYQSLLTKN-KARVIILMVWIVSGLTSFLPIQMHWYRA 176
                 **** .**.*::*** *:: *: *:   : :  . ::* :.* :*   *  *: :   .*

drd1_human       KPTSPSDGNATSLAETIDNCDSSLSRTYAISSSVISFYIPVAIMIVTYTRIYRIAQKQIR 226
drd2_human       ------DQNECIIAN----------PAFVVYSSIVSFYVPFIVTLLVYIKIYIVLRRRR- 220
adrb2_human      T-----HQEAINCYANETCCDFFTNQAYAIASSIVSFYVPLVIMVFVYSRVFQEAKRQLQ 231
                       . :                 ::.: **::***:*. : :..* :::   :::

drd1_human       RIAALERAAVHAKNCQTTTGNGKPVECSQPESSFKMSFKRETKVLKTLSVIMGVFVCCWL 286
drd2_human       ------------------------------------SQQKEKKATQMLAIVLGVFIICWL 235
adrb2_human      KIDKSEGRFHVQNLSQVEQDGRTG----HGLRRSSKFCLKEHKALKTLGIIMGTFTLCWL 287
                                                        :* *. : *.:::*.*  ***

drd1_human       PFFILNCILPFCGSGETQPFCIDSNTFDVFVWFGWANSSLNPIIY-AFNADFRKAFSTLL 345
drd2_human       PFFITHILNIHCDCN------IPPVLYSAFTWLGYVNSAVNPIIYTTFNIEFRKAFLKIL 289
adrb2_human      PFFIVNIVHVIQDNL------IRKEVYILLNWIGYVNSGFNPLIY-CRSPDFRIAFQELL 340
                 **** : :    .        *    :  : *:*:.**..**:**   . :** **  :*

drd1_human       GCYRLCPATNNAIETVSINNNGAAMFSSHHEPRGSISKECNLVYLIPHAVGSSEDLKKEE 405
drd2_human       HC---------------------------------------------------------- 291
adrb2_human      CLRRSSLKAYG--NGYSSNGNTGEQSGYHVEQEKENKLLCEDLPGTEDFVGHQGTVPSDN 398


drd1_human       AAGIARPLEKLSPALSVILDYDTDVSLEKIQPITQNGQHPT 446
drd2_human       -----------------------------------------
adrb2_human      IDSQGRNCSTNDSLL-------------------------- 413
```

Figure 1. The initial multiple sequence alignment between dopamine D_1 (drd1), D_2 (drd2) and the adrenergic β_2 receptors obtained from ClustalW.

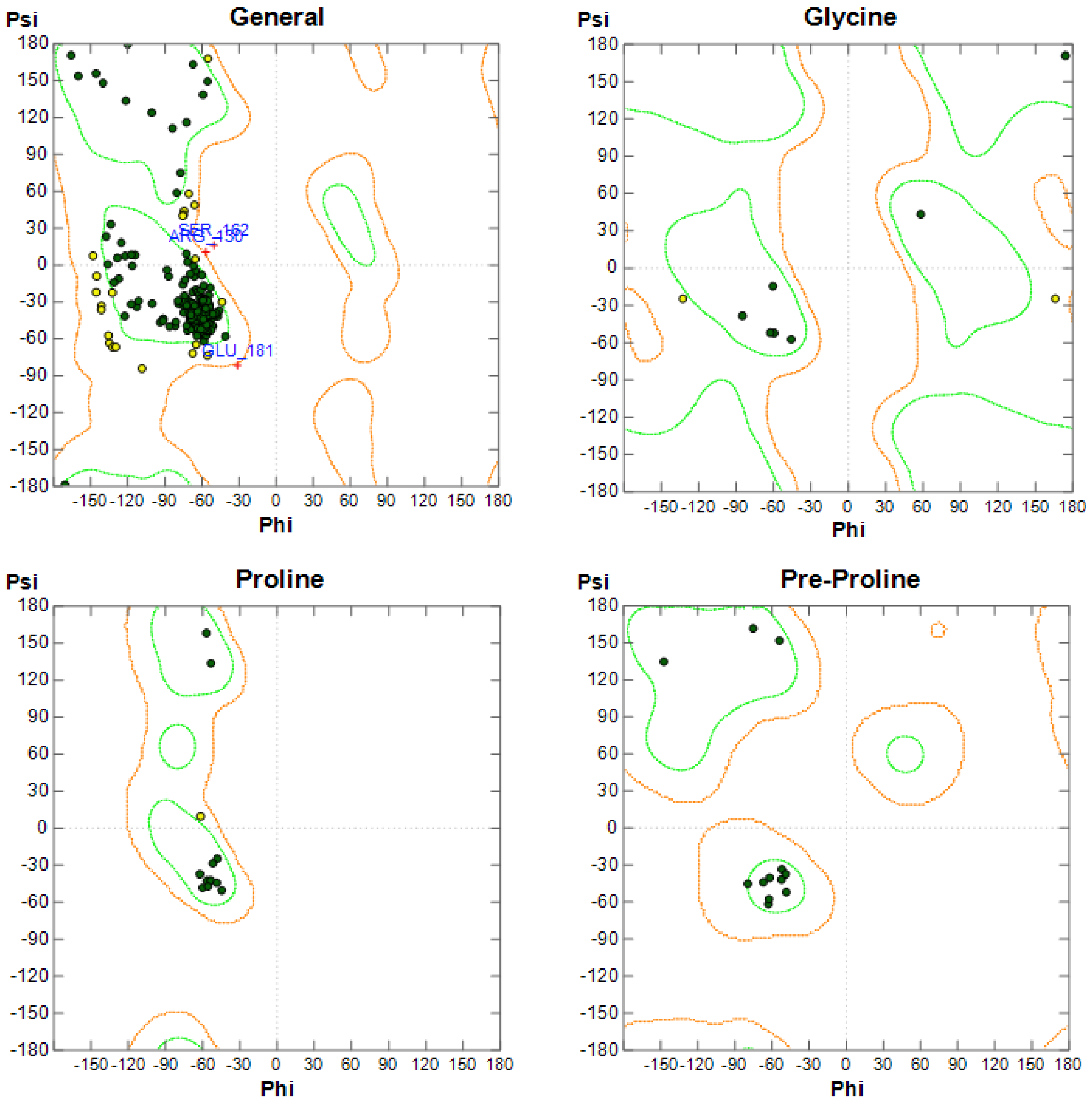

Figure 2. Ramachandran plots for glycines, prolines, pre-prolines and for general residues of the selected dopamine D_1 homology model. The contours indicate allowed (orange) and core (green) regions of φ and Ψ angles, and the filled green rings indicate amino acids within the core regions. The yellow rings indicate allowed regions. A red cross indicates outliers. The outliers Arg150, Ser162 and Glu181 are located in loops far apart from the binding site and are, therefore, considered to be acceptable.

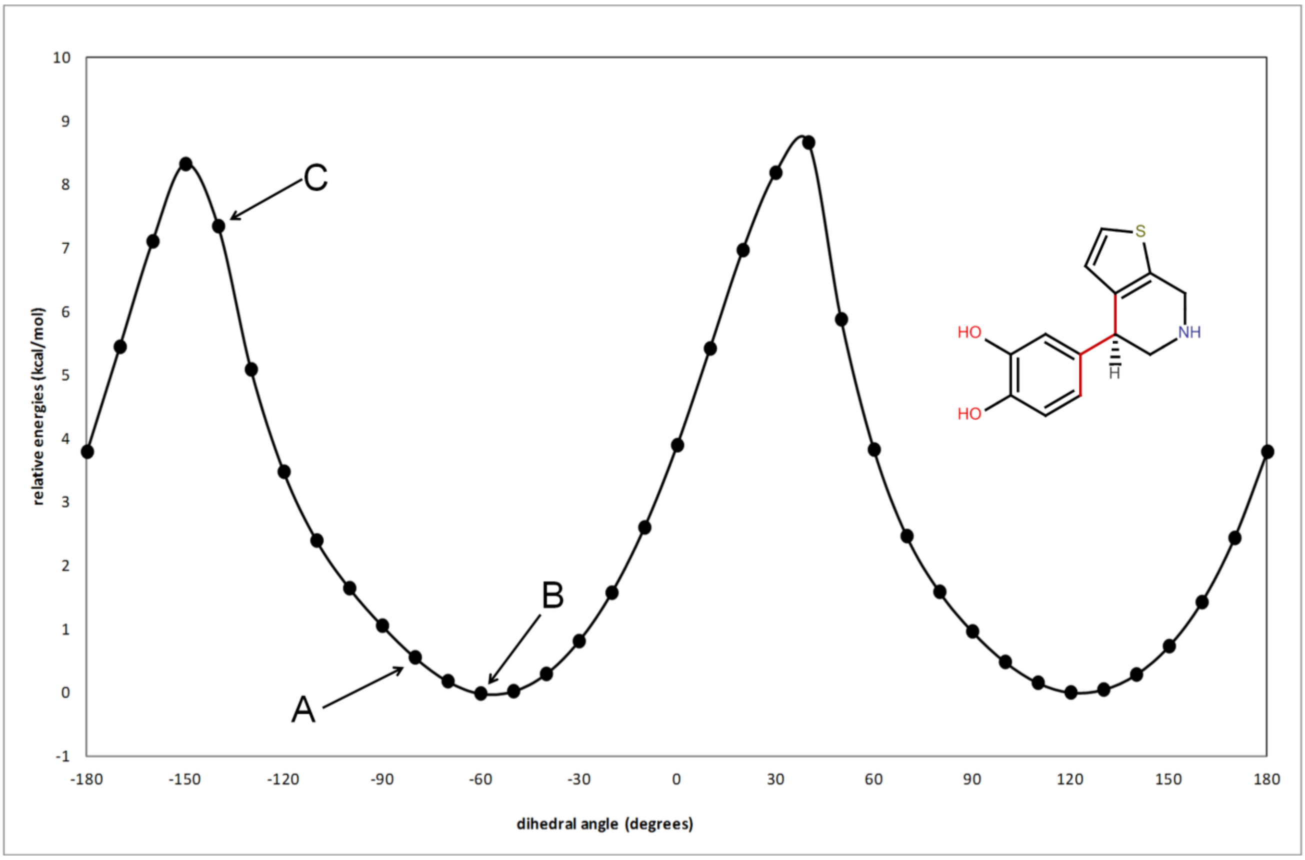

Figure 3. The dihedral angle energy profile for the bond connecting the two ring systems in SKF89626. The dihedral angle in the ligand is marked in red. A) The conformation of SKF89626 in the ligand/receptor complex with a relative energy of ~0.6 kcal. B) The global minimum. C) The conformer of SKF89626 just after the planarity flip.

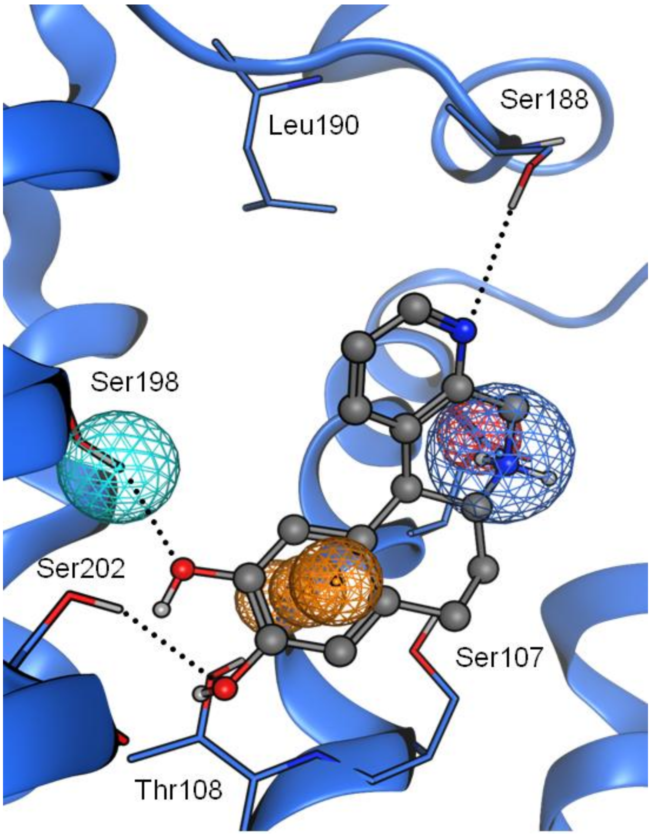

Figure 4. The DHX aza-analogue **1** forms a hydrogen bond with Ser188 in EC2 in the D_1 receptor model. The distance between the heavy atoms in the hydrogen bond is 4.3 Å and the angle (N---H-O(Ser188)) is 139°.
